# A National Department of Public Health

**Published:** 1890-04

**Authors:** 


### SELECTIONS.

### A National Department of Public Health.

As will be seen in the Notes and Comments of this number of the *Reporter*, it is proposed to establish a "Bureau of Animal Industry," for the suppression of contagious diseases among domestic animals, with rather broad powers, and to be under a chief who shall be a veterinary surgeon. It is to be hoped, of course, that some sensible way may be found to regulate matters of health in connection with cattle in the United States; but we doubt that this is the best way to get at the matter. In our opinion the subject should be committed to some of the existing departments, and made a part of a general system of sanitary supervision for the whole country. We regard it as unwise at this time to establish a bureau for the diseases of animals, which might be hard to force into the bounds of a larger scheme at a future date.

We would approve of legislation conferring on the Marine Hospital Service supervision over all matters of public health, in the hope that before long this would lead to the establishment of a Department of Public Health, fully equipped and placed on a footing equal to that of any of the existing departments. Certainly the health interests of the country are worthy of being represented in the Cabinet; and they should be so represented as soon as there is any general agreement among medical men as to the best way in which this representation can be carried out. A Department of Public Health seems to us to be one of the greatest needs of our great country to-day; and we invite our colleagues of the medical journals to discuss the means by which such a Department may be established and maintained as part of the National Government.—*Med. and Surg. Reporter.*

According to the statement of the registrar of the New York Board of Health, the greater ratio of deaths occur in old houses, modern houses being more conducive to health.

